# Genome Sequences of *Amycolatopsis orientalis* subsp. *orientalis* Strains DSM 43388 and DSM 46075

**DOI:** 10.1128/genomeA.00545-13

**Published:** 2013-08-01

**Authors:** Haeyoung Jeong, Young Mi Sim, Hyun Ju Kim, Yong-Jik Lee, Dong-Woo Lee, Si-Kyu Lim, Sang Jun Lee

**Affiliations:** Korean Bioinformation Center, Korea Research Institute of Bioscience and Technology (KRIBB), Daejeon, Republic of Koreaa; Infection and Immunity Research Center, KRIBB, Daejeon, Republic of Koreab; School of Applied Biosciences, Kyungpook National University, Daegu, Republic of Koreac; GenoTech Corporation, Daejeon, Republic of Koread

## Abstract

Strains of *Amycolatopsis orientalis* produce vancomycin or other related glycopeptide antibiotic compounds. Here we report the draft genome sequences of glycopeptide nonproducers *Amycolatopsis orientalis* subsp. *orientalis* DSM 43388 and DSM 46075. Their genome information will provide insights into the acquisition and regulation of glycopeptide antibiotic resistance genes.

## GENOME ANNOUNCEMENT

*Amycolatopsis orientalis* is known to produce glycopteptide antibiotics, including vancomycin (1) and other related antibiotic compounds such as orienticins (2), eremomycin (3), and chloroeremomycin (also known as A82846B and LY264826) (4). We previously reported the genome sequence of vancomycin-producing *Amycolatopsis orientalis* strain KCTC 9412^T^ (5). In this study, the strains DSM 43388 and DSM 46075 (ATCC 35164), which do not produce vancomycin and were obtained from the public culture collection, were chosen for sequencing with a view to understanding the diversification of glycopeptide antibiotic biosynthesis and resistance genes.

Cells were grown in tryptic soy broth containing wheat starch at 30°C. Cells were harvested and resuspended with 50 mM EDTA (pH 8.0) and were subsequently treated with achromopeptidase (5 mg/ml) and lysozyme (10 mg/ml) for cell disruption (6). Genomic DNA (gDNA) was purified by use of a gDNA isolation kit (Promega). Illumina library construction and genome sequencing were performed by the Human Derived Material Center in KRIBB, Daejeon, Republic of Korea, using an Illumina HiSeq 2000 system. A total of 2 × 101-nucleotide (nt) paired reads (47,213,684 and 49,370,206 bp) were produced for a total of 4.77 and 4.99 gb for DSM 43388 and DSM 46075, respectively.

Quality trimming of reads and *de novo* assembly were done using the CLC Genomics Workbench ver. 6.0.1 (CLC Bio). For DSM 43388, 197 contigs with a G+C content of 70.0% were obtained (total contig length, 8,920,384 bp; *N*_50_, 114,355 bp). The DSM 46075 assembly resulted in 182 contigs with a G+C content of 69.4% (total contig length, 9,445,329 bp; *N*_50_, 123,184 bp). The assembled sequences were automatically annotated using the RAST server (7). The numbers of protein-coding genes were 8,636 and 9,082.

Unexpectedly, the average nucleotide identities (ANI) of these two strains with the type strain KCTC 9412^T^, calculated by using the JSpecies program (8), were too low (76.6% and 76.5% for DSM 43388 and DSM 46075, respectively), suggesting these two strains should be reclassified to other species. The two strains are rather close to each other, with a high ANI value (>91%). Vancomycin biosynthetic gene clusters were not identified in the assemblies of DSM 43388 and DSM 46075. Vancomycin resistance genes *vanH, vanA*, and *vanX* (9) were located in the front of the vancomycin synthetic modules in the genome of KCTC9412^T^ (5). However, the *vanHAX* genes in DSM 46075 were located next to genes encoding a VanS/VanR two-component system, which might regulate the expression of glycopeptide antibiotic resistance genes.

### Nucleotide sequence accession numbers. 

These whole-genome shotgun projects have been deposited at DDBJ/EMBL/GenBank under the accession numbers ASXG00000000 and ASXH00000000 for DSM 43388 and DSM 46075, respectively. The versions described in this paper are ASXG01000000 and ASXH01000000.

## References

[1] Levine DP 2006 Vancomycin: a history. Clin. Infect. Dis. 42(Suppl 1):S5–S12 16323120 10.1086/491709

[2] Tsuji N Kobayashi M Kamigauchi T Yoshimura Y Terui Y 1988 New glycopeptide antibiotics. I. The structures of orienticins. J. Antibiot. 41:819–822 3403380 10.7164/antibiotics.41.819

[3] Berdnikova TF Tokareva NL Abramova EA Dokshina NIu Potapova NP 1988 The structure of the aglycon of eremomycin—a new antibiotic of the polycyclic glycopeptide group. Antibiot. Khimioter. 33:566–570 (In Russian.) 2848466

[4] Nagaraja R Berry DM Hunt AH Occolowitz JL Scahbel AA 1989 Conversion of antibiotic A82846B to Orienticin A and structural relationships of related antibiotics. J. Org. Chem. 54:983–886

[5] Jeong H Sim YM Kim HJ Lee D-W Lim S-K Lee SJ 2013 Genome sequence of the vancomycin-producing *Amycolatopsis orientalis* subsp. *orientalis* strain KCTC 9412^T^. Genome Announc. 1(3):e00408-13. 10.1128/genomeA.00408-13 PMC3695432 23814036

[6] Nikodinovic J Barrow KD Chuck JA 2003 High yield preparation of genomic DNA from *Streptomyces*. BioTechniques 35:932–936 14628665 10.2144/03355bm05

[7] Aziz RK Bartels D Best AA DeJongh M Disz T Edwards RA Formsma K Gerdes S Glass EM Kubal M Meyer F Olsen GJ Olson R Osterman AL Overbeek RA McNeil LK Paarmann D Paczian T Parrello B Pusch GD Reich C Stevens R Vassieva O Vonstein V Wilke A Zagnitko O 2008 The RAST server: rapid annotations using subsystems technology. BMC Genomics 9:75 18261238 10.1186/1471-2164-9-75 PMC2265698

[8] Richter M Rosselló-Móra R 2009 Shifting the genomic gold standard for the prokaryotic species definition. Proc. Natl. Acad. Sci. U. S. A. 106:19126–19131 19855009 10.1073/pnas.0906412106 PMC2776425

[9] Marshall CG Lessard IA Park I Wright GD 1998 Glycopeptide antibiotic resistance genes in glycopeptide-producing organisms. Antimicrob. Agents Chemother. 42:2215–2220 9736537 10.1128/aac.42.9.2215 PMC105782

